# In Vitro Efficacy of Foam Hand Sanitizers Against Enveloped and Non-Enveloped Viruses

**DOI:** 10.1007/s12560-025-09640-8

**Published:** 2025-04-03

**Authors:** Francis Torko, Kristen E. Gibson

**Affiliations:** Department of Food Science, Center for Food Safety, University of Arkansas System Division of Agriculture, University of Arkansas, 1371 West Altheimer Dr., Fayetteville, AR 72704 USA

**Keywords:** Bacteriophage, Tulane virus, MS2, Phi6, Surrogate, Foam hand sanitizer, Hand hygiene

## Abstract

Enveloped and non-enveloped virus transmission can occur via person-to-person contact and potentially through contaminated surfaces with human hands. Establishing the efficacy of hand sanitizers, including gel and foam formats, is crucial in reducing the transmission of viruses of human health concern, yet foam hand sanitizers are generally underexplored despite being widely used. Following American Society for Testing and Materials (ASTM) E1052-20, the efficacy of foam-based hand sanitizers—one non-alcohol-based hand sanitizer and four alcohol-based hand sanitizers with benzalkonium chloride and ethanol as active ingredients, respectively—were explored using bacteriophage phi6 (Φ6) as a surrogate for enveloped viruses and bacteriophage MS2 (*Emesvirus zinderi*) and Tulane virus (TuV) as surrogates for non-enveloped viruses. Significant differences in log reduction were observed among viruses (*P* ≤ 0.05). After a 10 s exposure, a 5.23 ± 1.64 log reduction was observed for Φ6 while MS2 remained resistant (0.04 ± 0.08 log_10_ reduction). Conversely, significant log reductions (*P* ≤ 0.05) were observed for TuV across all foam-based hand sanitizer products ranging from 0.07 ± 0.1 to 1.09 ± 0.22. An exposure time of 10 s (i.e., the typical rubbing time in real-world scenarios following hand sanitizer application) is likely sufficient for enveloped virus inactivation based on the inactivation of bacteriophage Φ6 by the tested commercially available products. However, longer exposure times or different hand sanitizer formulations may be required to achieve similar log reductions against non-enveloped viruses such as human norovirus based on the surrogates (MS2, TuV) tested.

## Introduction

Viral pathogens can be transmitted from an infected person to a susceptible person by direct person-to-person contact transmission or indirect contact transmission (Cortez & Weitz, 2013), such as contaminated environmental surfaces or food-contact surfaces. Surfaces contaminated with viral pathogens could lead to infection when pathogens are transferred to hands followed by self-inoculation and secondary transmission. Enveloped viruses including Severe Acute Respiratory Syndrome Coronavirus 2 (SARS-CoV-2), influenza, and other coronaviruses can be transferred to hands via contaminated surfaces. Baker and Gibson (2022a) reviewed the persistence of SARS-CoV-2 on surfaces, indicating a low but potential risk of fomite transmission. Similarly, Van Doremalen et al. (2020) and Choi et al. (2021) highlighted the potential for SARS-CoV-2 transmission via fomites. Thomas et al. (2014) established the survival of influenza A, spiked with respiratory mucus, on fingerpads for up to 30 min—a duration sufficient to allow for self-inoculation and potential person-to-person transmission.

Non-enveloped viruses such as hepatitis A virus, human rotaviruses, and human noroviruses (HuNoV) are transmitted via the fecal–oral route, where the virus may be picked up by the hands and subsequently ingested potentially causing infection, or via consumption of contaminated food or water. Anderson and Boehm (2021) established that bacteriophage MS2 (Emesvirus zinderi), a surrogate used to study HuNoV, can be easily transferred from surfaces to fingerpads, indicating the risk of virus transmission by hand via contaminated surfaces. Meanwhile, Xiao et al. (2017) established the likelihood of HuNoV transmission via fomites after re-examining a HuNoV outbreak in a hotel restaurant. The significance of HuNoV transmission by contact with contaminated fomites (de Graaf et al., 2017) cannot be underestimated. Contamination of healthcare surfaces contributes to HuNoV outbreaks (Weber et al., 2010), and for some time now, cruise ships have been recognized as settings where HuNoV outbreaks frequently occur (Gunn et al., 1980; Mouchtouri et al., 2024; Verhoef et al., 2008), with fomite transmission likely playing a crucial role in these outbreaks.

Upper respiratory infections, which are mainly caused by viruses such as influenza, coronavirus, rhinovirus, accounted for about 17.2 billion cases worldwide in 2019 (Jin et al., 2021). The global impact and health burden of these viruses are substantial. According to the World Health Organization (WHO) (World Health Organization, 2023), the coronavirus known as SARS-CoV-2, has led to more than 776 million confirmed cases and 7 million deaths from January 2020 to November 2024. According to Faramarzi et al. (2024), the global economic impact of COVID-19 disease accounted for approximately 86% of healthcare spending and 9.13% of GDP from 2020 to 2021 based on published data. Also, WHO (2023a) estimates one billion cases of influenza and a corresponding 290,000 to 650,000 deaths annually. A study by Bartsch et al. (2020) on the economic burden of HuNoV in the US indicated that HuNoV infections contributed $7.6 million and $165.3 million in direct medical costs and productivity losses annually, respectively. During the 2023 Joint Food and Agricultural Organization of the United Nations/WHO Expert Meeting on the microbiological risk assessment of viruses in foods, HuNoV was ranked as the number one cause of foodborne illness with an estimated 125 million annual cases and 35,000 deaths globally, with prepared food and contamination by food handlers being a significant contributor of HuNoV infections. This is supported by the US Centers for Disease Control and Prevention (CDC) which classified contamination of food, by food handlers, as a top factor in viral transmission causing foodborne outbreaks (CDC, 2023).

Hand hygiene is recognized as the foremost strategy for significantly reducing infectious agents on the hands, thereby minimizing their transfer to susceptible individuals or surfaces (CDC, 2021). Both handwashing with soap and running water as well as the use of hand sanitizers are part of hand hygiene strategies. The CDC advocates for handwashing with soap and water, especially when hands are visibly soiled. However, in the absence of soap and water, the use of a hand sanitizer—a liquid, gel, or foam product designed to reduce infectious organisms such as viruses, bacteria, and fungi on the hands (Gold et al., 2018)—is recommended. Evaluating the efficacy of hand sanitizers in controlling both enveloped and non-enveloped viruses helps guide public health measures. However, many efficacy studies have been conducted on gel-based hand sanitizers (Grayson et al., 2009; Siddharta et al., 2017; Stauffer et al., 2013), while the foaming hand sanitizers are less explored.

Most hand sanitizers contain either ethanol or isopropanol as their active ingredient and are generally referred to as alcohol-based hand sanitizers (ABHSs). A typical active ingredient in non-alcohol-based hand sanitizers (NABHSs) is benzalkonium chloride (BZK), a quaternary ammonium compound. Unlike foam-based hand sanitizers—products which contain foamitizers that entrap air to form bubbles (Fameau et al., 2021)—previous research has examined the actions of hand sanitizers, particularly gel-based ones which are viscous products containing thickening agents (Fallica et al., 2021)—on the reduction of infectious virus particles, often focusing on the concentrations of active ingredients such as ethanol or BZK (Kampf, 2018; Park et al., 2010; Tung et al., 2013). Although higher concentrations of active ingredients are generally associated with greater virus reduction, recent studies suggest that the inactivation of viruses may depend on the overall formulation, including the inactive ingredients, rather than solely on the active ingredients (Escudero-Abarca et al., 2022; Siddharta et al., 2017; Suchomel et al., 2020). These findings highlight the importance of considering the complete formulation of hand sanitizers in efficacy studies, rather than focusing exclusively on the active ingredients. Also, while limited studies are available on the comparative efficacy of hand sanitizers, considering factors such as formats or delivery mode (Grayson et al., 2009; Larson et al., 2012), research on the efficacy of ABHS and NABHS against bacteria is well-documented (Booq et al., 2021; D’Antonio et al., 2010; Fendler et al., 2002; Gold et al., 2018; Jain et al., 2016; Kampf et al., 2010).

As indicated, research on the efficacy of foam-based hand sanitizers against viruses is limited. Therefore, this in vitro study aimed to compare the effectiveness of alcohol and non-alcohol-based foaming hand sanitizers with varying concentrations of active ingredients (ethanol and BZK) and different formulations of inactive ingredients. The impact of foam-based hand sanitizer type on the log reduction of enveloped and non-enveloped viruses was investigated while considering the effects of organic matter and inoculum concentration on the reduction of infectious virus particles. Understanding these factors is crucial for the selection of effective hand hygiene strategies to potentially reduce the burden of disease due to pathogenic viral agents.

## Materials and Methods

### Bacterial Host Cultivation and Bacteriophage Propagation

Phi6 (Φ6) and MS2 (ATCC 15597-B1; American Type Culture Collection, Manassas, VA) bacteriophage (phage)—surrogates for the study of enveloped and non-enveloped viruses, respectively—were propagated using previously described methods (Baker & Gibson, 2022b; Gibson et al., 2012). Briefly, Φ6 was grown in *Pseudomonas syringae* pathovar *phaseolicola* (Pph) on lysogeny agar/broth (LC; 10 g/L NaCl, 10 g/L tryptone, 5 g/L yeast extract, pH 7.5) while MS2 was cultivated in *Escherichia coli* C3000 (ATCC 15597; ATCC) using tryptic soy broth (TSB) (30 g/L of TSB; Becton, Dickson and Company, Sparks, Maryland) and agar (TSA) (40 g/L of TSA; Becton, Dickson and Company, Sparks, Maryland). Φ6 and Pph were kindly provided by Dr. Sylvain Moineau at Université Laval, Québec, Canada. Phage stock was produced via the double agar overlay assay (DAL). For this, a single colony of Pph or *E. coli* was cultured overnight in 25 mL of LC broth or TSB. Subsequently, 250 μL of Pph or 50 μL of *E. coli* C3000 culture was mixed with 100 μL of phage stock (~ 10 log_10_ PFU/mL) in 5 mL of respective soft agar, poured onto corresponding plates, and incubated overnight at 25 °C (Φ6) or 37 °C (MS2). Plates showing lysis were harvested, filtered, and stored at 4 °C or −20 °C (Φ6) and −80 °C (MS2) for later use.

### LLC-MK2 Cultivation and Tulane Virus Propagation

Tulane virus (TuV) was used as an alternative surrogate to study non-enveloped viruses and was obtained from Dr. Jason Jiang (Cincinnati Children’s Hospital Medical Center, Cincinnati, Ohio). The cultivation of LLC-MK2 cells (ATCC CCL-7; American Type Culture Collection, Manassas, VA) and TuV propagation were performed according to methods described previously by Arthur and Gibson (2015) with modifications. After TuV propagation and harvesting, the supernatant containing virus was filtered via a 0.22 µm pore size syringe filter pretreated with 1% Tween 80 and then washed with 1× PBS. Following filtration, TuV titration was performed as previously described (Arthur & Gibson, 2015). Briefly, 6-well plates were seeded with 2 × 10^5^ LLC-MK2 cells and incubated at 5% CO_2_ and 37 °C. 500 μL of serially diluted virus-sanitizer suspension were then added to 6-well plates, of 85–100% confluent LLC-MK2 cell monolayer, in duplicates. Plates were incubated at 37 °C, 5% CO_2_ with gentle rocking for 1 h. Following incubation, samples were aspirated and 2 mL of 3% low-melting agarose (NuSieve® GTG Agarose; Lonza, Basel, Switzerland) was added over cell monolayers and incubated at 37 °C, 5% CO_2_ for 4 days. Plaques were then stained with 6% neutral red (Sigma-Aldrich, St Louis, Missouri), incubated for 3 h, and visualized to determine the concentration of the recovered virus.

### Organic Matter Preparation

A tripartite solution was prepared as a source of organic matter during virus exposure to hand sanitizers. Briefly, the tripartite solution was prepared according to American Society for Testing and Materials (ASTM) E2011-21 (Standard Test Method for Evaluation of Hygienic Handwash and Handrub Formulations for Virus-Eliminating Activity Using the Entire Hand) by adding 0.5 g of yeast extract to 10 mL of 1× PBS, 0.5 g of bovine serum albumin (BSA) to 10 mL of 1× PBS, and 0.04 g of bovine mucin to 10 mL of 1× PBS (ASTM, 2021). The stock solutions were prepared separately and filtered using a 0.22 µm pore size syringe filter. All stock solution components were stored at 4 °C until use.

### Hand Sanitizer Products and Neutralizer Effectiveness/Toxicity

The efficacy of five commercially available foam hand sanitizers was explored in this study. One of the five products was a NABHS (Global Industrial™ Foam Hand Sanitizer Alcohol Free, Global Industrial, Bufor, GA) while the remaining four were ABHSs: Global Industrial™ Foam Hand Sanitizer 62% Alcohol (Global Industrial, Bufor, GA), Tork® Premium Alcohol Foam Hand Sanitizer (TORK, Philadelphia, PA), PURELL® Advanced Hand Sanitizer Green Certified Foam (Gojo Industries, Irving, TX), and InstantFOAM Complete (SCJ Professional, Charlotte, NC). Table 1 lists the selected hand sanitizers with their corresponding active and inactive ingredients, as well as their pH values. Dey/Engley (D/E) Neutralizing Broth (BD Life Sciences, Franklin Lakes, NJ) was used in this study, and ASTM E1052-20 (Standard Practice to Assess the Activity of Microbicides against Viruses in Suspension) (ASTM, 2020) was used to determine its effectiveness and cytotoxicity. For the neutralizer effectiveness test, about 2.24 × 10^3^ PFU of virus (~ 100 µL) was added to 900 μL of neutralizer. One hundred microliters of hand sanitizer were added to the neutralizer-virus suspension, and the DAL method for Φ6 and MS2 was performed afterward as described previously (Baker and Gibson, 2022b; Gibson et al., 2012) while a plaque assay was performed for TuV as described earlier. For the neutralizer cytotoxicity test, 100 μL of 1 × PBS was added to neutralizer-virus suspension instead of hand sanitizer. Comparable results of neutralizer effectiveness and cytotoxicity to the baseline virus control (virus suspension in LC) concentration were achieved to ascertain the effectiveness and non-toxicity of the neutralizer to viruses. The cytotoxicity of the neutralizer alone, neutralized hand sanitizers, and hand sanitizers alone to host cells was tested. Briefly, for the phages host cells, 100 μL of neutralizer alone, neutralized hand sanitizers, or hand sanitizers alone were combined with 250 μL of Pph in LC soft agar or 50 μL of *E. coli* C3000 in TSA soft agar which was representative of host cell exposures during the in vitro suspension assay. As a host control, 250 μL of Pph or 50 μL of *E. coli* C3000 were suspended in an LC or TSA soft agar, respectively, and plated to compare the bacterial lawn appearance in the host control to the results of the cytotoxicity tests. For TuV host cells (LLC-MK2), 500 μL of neutralizer, neutralized hand sanitizers, hand sanitizers, and Opti-MEM medium (control) were each added to 85–100% confluent cell monolayer in a 6-well plate, rocked at 37 °C and 5% CO_2_ for 1 h, and incubated for 4 days after which the integrity of the cell monolayer was observed. Experiments were performed in the presence and absence of test organisms to determine the susceptibility of host cells to infection.

**Table 1:** List of selected hand sanitizer formulations utilized in the present study

Product	Active ingredient	Inactive ingredients	pH
A	0.13% BZK	Water, Propylene Glycol, Cocamidopropyl Betaine, Aloe Barbadensis Leaf Juice, Tocopheryl Acetate (Vitamin E), PEG-7 Glyceryl Cocoate, Fragrance, Phenoxyethanol, Tetrasodium EDTA	5.61 ± 0.13
B	62% ethanol	Water, PEG-10Dimethicone, Glycerin, Isopropyl Myristate, Polyquaternium-11, Disodium EDTA, Aloe Barbadensis Leaf Juice, Tocopheryl Acetate	5.65 ± 0.14
C	70% ethanol	Water (Aqua), Isopropyl Alcohol, PEG-12 Dimethicone, Caprylyl Glycol, Glycerin, Isopropyl Myristate, Tocopheryl Acetate	6.18 ± 0.02
D	70% ethanol	Water/Eau/Aqua, Glycerin, Carbomer, Glycereth-7 triacetate, Triethanolamine, Tocopheryl acetate	6.2 ± 0.05
E	85% ethanol	Aqua, DL-Panthenol, Bis-PEG-12 Dimethicone, Dihydroxypropyl PEG-5 Linoleammonium Chloride, PEG-200 Hydrogenated, Glyceryl Palmitate, PEG-7 Glyceryl Cocoate, Coco-glucoside, Glyceryl Oleate, Citric Acid	6.67 ± 0.09

*BZK* benzalkonium chloride

### In Vitro* Suspension Test*

Tulane virus was suspended in Opti-MEM maintenance medium, supplemented with 2% fetal bovine serum (FBS), in the presence or absence of OM and used for in vitro experiments while Φ6 and MS2 were prepared as a cocktail, suspended in LC broth in the presence or absence of OM. Virus suspensions were prepared for high inoculum concentration (3 × 10^7^ PFU/mL) and low inoculum concentration (3 × 10^4^ PFU/mL). Due to challenges producing high titer value for TuV, high inoculum suspension for TuV was prepared at 9.5 × 10^6^ PFU/mL instead of 3 × 10^7^ PFU/mL. The virus suspension with OM contained 5% BSA, 20% bovine mucin, and 7% yeast extract in its final volume. Hand sanitizer products were dispensed into 50 mL centrifuge tubes using an Alpine automatic hand sanitizer dispenser (Alpine Industries Inc., Irvington, New Jersey), then tightly sealed and allowed to liquefy (from foam format) before being used in the experiment. The ASTM E1052-20 standard was employed for in vitro tests with modifications to inoculum and hand sanitizer volume parts (ASTM, 2020). Approximately 200 μL of the virus-OM suspension was added to 400 μL (approximate liquid volume per unit dispensed after foam hand sanitizer liquefies) of hand sanitizer to obtain 7 log_10_ PFU/mL (6.5 log_10_ PFU/mL for TuV) or 4 log_10_ PFU/mL of virus exposed to hand sanitizer for high and low inoculum concentration levels respectively. For control, the virus mixture was exposed to 400 μL of 1× PBS instead. The mixture was allowed to stand for an exposure time of 10 s followed by serial dilution. The first dilution (twofold or fivefold) was performed in D/E neutralizing broth to halt the microbicidal activity of the hand sanitizer products similar to ASTM E1054-22 with modifications. For Φ6 and MS2, the dilutions were then plated by DAL as described previously to determine the concentrations of recovered virus overnight while plaque assay was performed for TuV as outlined earlier.

### Data Analysis

A factorial experimental design was used for this study where each experimental combination was performed in triplicate. Samples were plated in duplicates, and the results were averaged to obtain a single value for each data point. All experiments were performed in a random order to control for potential lurking variables (e.g., virus aggregation) that might have an impact on the results of the experiment, but which were not investigated. Two models of analysis of variance (ANOVA) were constructed: one incorporating only the main effects of the variables explored, and the other including both main effects and two-way interactions of the independent factors. Upon comparison of both models, the model with two-way interactions demonstrated significant contributions, indicating that these interactions were important to include in the model. Therefore, the model with two-way interactions was selected for further analysis. Where necessary, a one-sample Student’s t-test was computed to determine whether the log reductions for the individual products were statistically different from zero. All PFU/mL values were log_10_ transformed into log_10_ PFU/mL. All analyses were performed using the R statistical software version 4.2.2, and a statistical significance threshold was set at *P* ≤ 0.05. The response variable, log reduction, was computed by subtracting the log_10_ PFU/mL value of treatment from the log_10_ PFU/mL of control. Where the difference was less than 0 (negative), 0 log_10_ PFU/mL was assigned.

## Results

### Overall Virucidal Efficacy

**Fig. 1:**
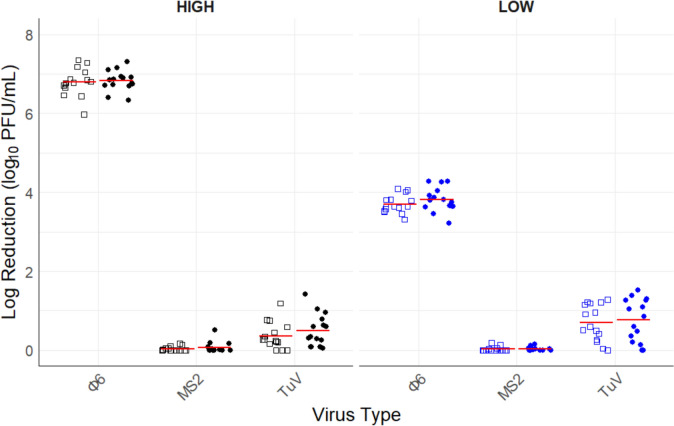
Overall log reduction achieved by all test products against enveloped, Φ6, and non-enveloped viruses, TuV and MS2, at high (black) and low (blue) initial inoculum concentrations for an exposure time of 10 s in the presence (dot) and absence (open square) of organic matter. Each data point represents a log_10_ PFU/mL of difference between treatment and control values, while the red line represents average of the factor levels. Φ6, bacteriophage phi6; MS2, *Emesvirus zinderi*; TuV, Tulane virus (Color figure online)

**Fig. 2:**
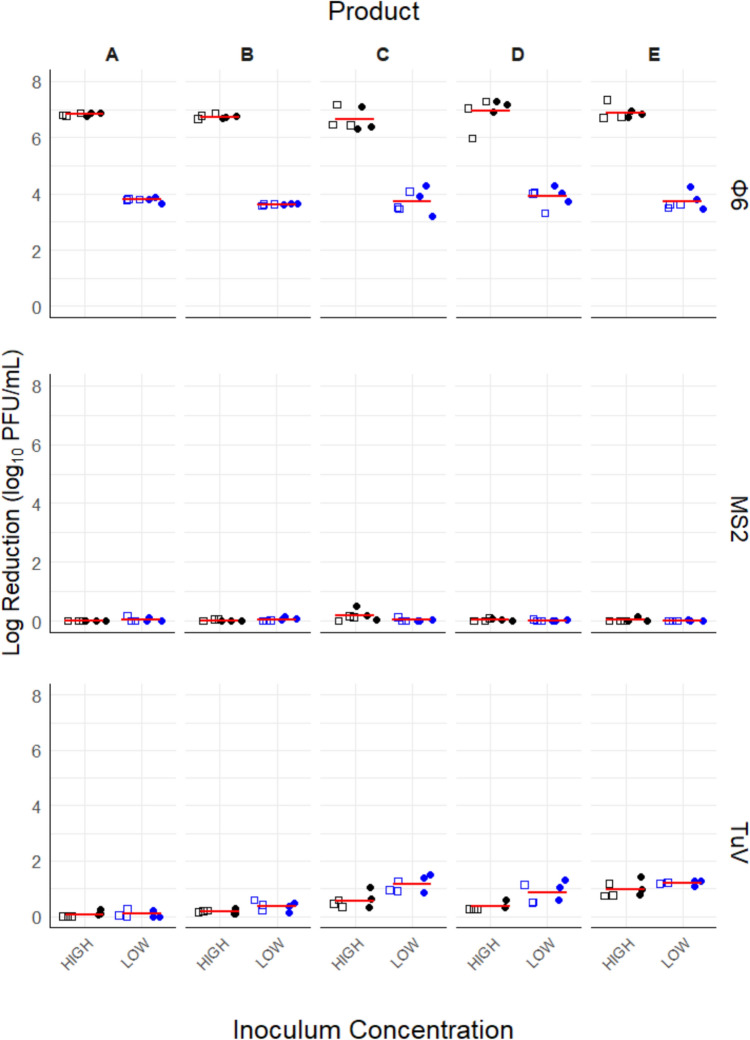
Efficacy of test products against TuV, MS2, and Φ6 at high and low initial inoculum concentrations, with a 10 s exposure time, in the presence (blue dot) and absence (open square) of organic matter. Each data point represents the log_10_ PFU/mL difference between treatment and control values while the red line represents the mean. Φ6, bacteriophage phi6; MS2, *Emesvirus zinderi*; TuV, Tulane virus (Color figure online)

Significant differences in log reduction were found between all viruses and inoculum concentrations. The highest overall log reduction of 5.23 ± 1.64 was observed for Φ6, followed by TuV and MS2. Mean log reductions for Φ6 and TuV were significantly different from zero (no log reduction) for all test products. At high inoculum concentration, log reduction of Φ6 (6.81 ± 0.3) was significantly higher (*P* ≤ 0.05) than at low inoculum concentration which yielded a log reduction of 3.66 ± 0.52 (Fig. 1). The average log reduction of Φ6 was the same value as the amount of virus exposed to the test products. Thus, there was inactivation of Φ6 below the limit of detection (0.18 log_10_) at both low and high inoculum concentrations. Both non-enveloped viruses, TuV and MS2, showed significant differences in log reduction. MS2 had a negligible mean log reduction of 0.04 ± 0.08 across all factors, with no statistically significant effects observed for inoculum concentrations (*P* = 0.285). TuV, on the other hand, had a higher log reduction (0.58 ± 0.46) compared to MS2. In addition, TuV showed significant differences among inoculum concentrations (*P* ≤ 0.05) with high and low inoculum concentrations recording 0.43 ± 0.37 and 0.73 ± 0.5, respectively. The presence or absence of organic matter did not impact (*P* = 0.412) the inactivation of the viruses (Figs. 1 and 2). Significant two-way interactions were observed between products and virus type, and inoculum concentration and virus type (*P* ≤ 0.05), suggesting that virus log reduction depends on the hand sanitizer used and the virus concentration exposed to the hand sanitizer (Fig. 2).

### Product Efficacy against Enveloped and Non-enveloped Viruses

ANOVA output revealed significant differences among products (*P* ≤ 0.05). Post hoc Tukey analysis showed no significant difference between products A and B or among products C, D, and E, but A and B were significantly different overall from C, D, and E (refer to Table 1 for products). Overall, product E (2.14 ± 2.5) had the highest log reduction followed by products C (2.05 ± 2.45), D (2.02 ± 2.63), B (1.83 ± 2.58), and A (1.8 ± 2.67) across all factor levels. Examining the efficacy of the products against the individual viruses, there were no significant differences among products for Φ6 (*P* = 0.3) and MS2 (*P* = 0.07). On the contrary, the efficacy of the products against TuV was significantly different (*P* ≤ 0.05). TuV, log reductions achieved by products A and B were significantly lower than products C, D, and E. Moreover, product D had a significantly lower log reduction when compared to products C and E. The average TuV log reductions were 0.07 ± 0.1 (A), 0.27 ± 0.16 (B), 0.86 ± 0.4 (C), 0.62 ± 0.36 (D), and 1.09 ± 0.22 (E) as shown in Fig. 2. While there was an overall significantly lower TuV reduction, as reported early on, for high concentration than low concentration (Fig. 2), further analysis revealed that not all products had significant differences in log reduction between high and low inoculum concentrations. Specifically, there was no statistical difference between high and low inoculum concentrations for products A, B, and E. However, differences were observed for products C and D, with the lower concentrations having higher log reduction than the high concentrations. A significant interaction between products and inoculum concentration for TuV suggests that log reduction by a hand sanitizer product depends on the TuV concentration exposed to the product.

### Neutralization Assay

For the neutralization assay, no statistically significant differences were observed in neutralization effectiveness, cytotoxicity, and control for Φ6 (*P* = 0.72) and TuV (*P* = 0.81). The virus particles recovered for both Φ6 and TuV were comparable across effectiveness, cytotoxicity, and control assays (Table 2). While neutralization effectiveness for MS2 was significantly higher (*P* ≤ 0.05) than cytotoxicity and control, this may be due to experimental errors. Overall, the neutralization assay results confirmed the effectiveness and non-toxicity of neutralizer to viruses. For host cells cytotoxicity tests, a bacterial lawn appearance of Pph and *E. coli* C3000 was observed for the neutralizer, neutralized products, and test products with no evident cytopathic effects. In LLC-MK2 cells, morphological changes were observed when cells were exposed to hand sanitizer products alone, and cells were not susceptible to TuV infection. When exposed to neutralizer alone or neutralized samples, minor cellular changes were noted; however, cells remained susceptible to TuV infection as indicated by previously reported PFU values.

**Table 2:** Mean log_10_ PFU of neutralization assays for Φ6, MS2, and TuV across all products

Assay	Φ6	MS2	TuV
Effectiveness	3.28 ± 0.1	3.84 ± 0.13	3.13 ± 0.04
Cytotoxicity	3.33 ± 0.13	3.77 ± 0.16	3.06 ± 0.03
Control	3.35 ± 0.16	3.84 ± 0.17	3.05 ± 0.02

*Φ6* bacteriophage phi6, *MS2 Emesvirus zinderi*, *TuV* Tulane virus

## Discussion

This study aimed to compare the efficacy of four alcohol (ethanol-based) and one non-alcohol (BZK-based) foaming hand sanitizers with different active ingredients concentrations and different inactive ingredients formulations against enveloped and non-enveloped virus surrogates in the presence or absence of organic matter and at high or low inoculum concentrations. A 10 s exposure time was sufficient to completely inactivate the enveloped virus surrogate, Φ6, by all the test products. However, minimal inactivation was observed for the non-enveloped virus surrogates, TuV and MS2.

Regarding enveloped viruses, numerous studies have demonstrated the efficacy of gel-based ABHS (Grayson et al., 2009; Siddharta et al., 2017) and ethanol solutions against enveloped viruses (Kampf, 2018; Park et al., 2010). However, there is limited research on foam-based hand sanitizers, which are widely used. Here, the inactivation of Φ6 to below the LOD across all tested products, regardless of organic matter presence and virus concentration aligns with Kampf (2018), who found that 80% ethanol was highly effective against 21 tested enveloped viruses within 30 s but less effective against non-enveloped viruses and surrogates such as feline calicivirus (FCV), polyomavirus SV40, and hepatitis A virus. The high susceptibility of Φ6 could be due to the high sensitivity of its outer lipid bilayer membrane (Pinon & Vialette, 2019) which is disrupted by the hand sanitizers.

As reported earlier, organic matter did not protect Φ6 from inactivation, consistent with findings by Ogilvie et al. (2021), where quaternary ammonium-based products effectively reduced SARS-CoV-2 (> 2 log_10_ reduction) with and without organic load within 15 s. Though it appeared that without soil load, log reductions were slightly higher, the authors did not report any statistical significance. Rabenau et al. (2005) also reported no protective effect of organic matter on the inactivation of SARS-CoV by four ABHS and three BZK-containing disinfectants. The study results indicated that all four sanitizers completely inactivated SARS-CoV below the LOD (reduction factor of ≥ 4.3 to ≥ 5.5) within 30 s. In addition, at both inoculum concentrations, there was no protective impact on the log reduction of Φ6 in our study. Bangiyev et al. (2021) found that higher concentrations protected Φ6 from environmental decay in saline solution at 4 °C but had no impact on Φ6 decay in LC growth medium (pH adjusted to 7.0) inoculated on plastic surfaces. This similarity in the present study may be due to the suspension of Φ6 in LC during our cocktail preparation.

For non-enveloped viruses, MS2 and TuV were selected as surrogates to better understand the potential inactivation of HuNoV during exposure to foam-based hand sanitizers. MS2 is an established surrogate for the study of non-enveloped viruses, such as HuNoV (Park & Sobsey, 2011). TuV, an alternative surrogate for the study of HuNoV, is selected based on its ability to recognize the same histo-blood group antigen receptor as HuNoV. Additionally, being part of the *Caliciviridae* family like HuNoV, TuV shares similar structural characteristics, making it a suitable model for studying the inactivation mechanisms of HuNoV (Huang et al., 2022; Sun et al., 2024; Taligrot et al., 2022). This is particularly important given the challenges associated with culturing HuNoV using cell- and animal-based models (Chandran & Gibson, 2024). Additionally, while the susceptibility of non-enveloped virus surrogates such as MS2, FCV, and murine norovirus (MNV) to chemical sanitizers is well-documented, less is known about the inactivation of TuV.

In this study, MS2 was more resistant to inactivation than TuV, highlighting differences in susceptibility of non-enveloped viruses. This aligns with previous research showing variability in non-enveloped virus inactivation. As mentioned earlier, TuV and MS2, as well as other surrogates including MNV and FCV, have been used to study non-enveloped viruses such as HuNoV on surfaces (Chiu et al., 2015; Park & Sobsey, 2011) and in suspension (Park et al., 2010; Tung et al., 2013). Though limited research exists on the inactivation of MS2 and other non-enveloped virus surrogates by foam hand sanitizers, studies have tested the efficacy of chemical sanitizers against these surrogates, especially FCV, MS2, and MNV. Su and D’Souza (2012) reported that BZK solutions at different concentrations (0.2, 0.5, and 1 mg/mL) completely inactivated FCV and MNV at approximately 5 log_10_ PFU/mL in a suspension test, while MS2 was only reduced by 1.47 to 1.8 log_10_ PFU/mL. At higher inoculum concentrations (approximately 7 log_10_ PFU/mL), reductions for FCV and MNV ranged from 2.87 to 3.25 log_10_ PFU/mL and 1.55 to 2.75 log_10_ PFU/mL, respectively, while MS2 had an approximate 2 log_10_ PFU/mL reduction. Numerous studies have evaluated the efficacy of a variety of chemical disinfectants (e.g., sodium hypochlorite, peracetic acid [PAA]) against non-enveloped virus surrogates demonstrating a wide range of virucidal susceptibilities in suspension (Fuzawa et al., 2020) and contact surface (Park & Sobsey, 2011). Notably, MS2 oftentimes demonstrated greater resistance to inactivation compared with FCV and MNV. The above studies emphasize the varying susceptibilities of non-enveloped viruses to inactivation as was observed in the current study. The significant differences in the efficacy of the test products against TuV and MS2 may be due to the different structural compositions of the protein capsids of both viruses which impact their susceptibility to inactivation (Narula et al., 2024).

For TuV, the test products showed varying virucidal activity. Product E, containing 85% w/w ethanol, achieved the highest log reduction after a 10 s exposure while the NABHS product, with 0.13% BZK, had the lowest log reduction. These results align with Escudero-Abarca et al. (2022) where a gel-based hand sanitizer with 85% v/v ethanol was observed to reduce HuNoV GII.4 by 3.3 ± 0.3 log_10_ genome equivalent copies (GEC) during a 30 s in vivo finger pad experiment, while a foam-based NABHS with 0.1% BZK achieved a reduction of 0.3 ± 0.2 log_10_ GEC during the same exposure duration. Although the trend is similar to the present study, the greater virus reduction reported by Escudero-Abarca and coauthors could be due to the hand sanitizer format, formulation (i.e., inactive ingredients), and longer exposure time compared to the 10 s exposure utilized here. Moreover, the significant differences in the inactivation of TuV by products E (ethanol-based) and A (BZK-based) could be due to the differences in the inactivation mechanisms of ethanol and BZK. Ethanol disrupts viral protein structures by forming polar bonds with the protein capsid causing denaturation. In contrast, BZK primarily disrupts viral membranes by interrupting with charge distribution, a mechanism that is highly effective against enveloped viruses (Ishikawa et al., 2002) but less effective against non-enveloped viruses.

Importantly, Escudero-Abarca et al. (2022) emphasized that the overall formulation, not just the active ingredient concentration, determines hand sanitizer efficacy. The study authors’ conclusion is based on comparable inactivation of HuNoV when exposed to a product with a high ethanol concentration (85% v/v) and those with lower concentrations (68–70% v/v). In the present study, products C and E, with ethanol concentrations of 70% v/v and 85% w/w, respectively, showed no significant differences in TuV log reductions. However, product D, also containing 70% v/v ethanol, had significantly lower reductions than product C (also 70% v/v), further highlighting the importance of both active and inactive ingredients in the efficacy of hand sanitizers for virus reduction.

In the present research, inoculum concentration also influenced TuV log reduction; however, not all test products showed significant differences between low and high TuV concentrations. Products C and D (both formulated with 70% v/v ethanol) had significantly lower log reduction at high TuV concentrations compared to low concentrations. In contrast, other products did not show such significant variations between high and low virus concentrations, suggesting that the efficacy of products C and D may be compromised at higher viral loads. The reduced log reduction of TuV at high concentration is similar to observations made by Su and D’Souza (2012), as previously stated, where FCV and MNV had lower log reductions at a higher inoculum concentration than at a lower inoculum concentration. This may be due to protective effects offered by the higher number of viruses present.

There was no impact of organic matter on the inactivation of TuV and MS2, similar to Φ6. Previous studies (Ionidis et al., 2016; Jubinville et al. 2021; Magulski et al., 2009) have examined the impact of organic matter on virus inactivation. Jubinville et al. (2021) evaluated the effect of organic matter sources (e.g., ASTM tripartite organic load, FBS, artificial feces, real fecal matter) on MNV inactivation when exposed to chemical sanitizers in suspension. The study authors established that organic matter provided some protection of MNV against PAA; however, the same protective effects were not seen when MNV was exposed to sodium hypochlorite. Jubinville et al. (2021) acknowledged that the impact of organic matter on MNV inactivation may be dependent on the treatment method used. In the present study, there was no significant impact of organic matter on log reduction for TuV and MS2 by the test products which may be attributed to the disinfection method utilized, specifically the use of foam-based hand sanitizer, as suggested by Jubinville et al. (2021).

The overall low log reduction observed for TuV and MS2 could be due to aggregation of viral particles which may have protected virions against inactivation as observed by Fuzawa et al. (2020) and Mattle et al. (2011). Fuzawa et al. (2020) observed an increase in TuV aggregates at pH 3 over a 30 min duration based on an aggregate size increase from 250 to 1100 nm, whereas RV aggregates maintained a 200 nm size. RV was more susceptible to inactivation by PAA than TuV which the authors, in part, attributed to reduced aggregation in RV, allowing greater surface area interaction with PAA compared to TuV. Similarly, Mattle et al. (2011) used MS2 to investigate the role of virus aggregation on inactivation by PAA, finding that larger aggregates and higher PAA concentrations increased the inhibitory effect of aggregation on disinfection. The authors also noted that the inactivation rate was diminished by two- to six-fold due to aggregation. In our study, although we did not investigate what impact virus aggregation could have on inactivation by the test products, the reduced inactivation of both non-enveloped virus surrogates may have been influenced by viral aggregate formation. To mitigate such effects, the randomization of experimental conditions was implemented. However, future studies should further investigate the impact of viral aggregates on the efficacy of foam hand sanitizers. Beyond virus aggregation, the high resistance to inactivation by TuV and MS2 may be due to their high protein capsid stability (Narula et al., 2024).

Although organic matter did not affect the efficacy of the hand sanitizer products against individual viruses, another factor that may have influenced viral inactivation is the differences in inoculum suspension matrix (Φ6 and MS2 suspended in LC, TuV suspended in Opti-MEM supplemented with 2% FBS) may have influenced the observed log reductions achieved against the viruses. This aligns with findings by Jubinville et al. (2021), where different inoculum suspension matrices affected MNV inactivation by chemical sanitizers. Similarly, Bangiyev et al. (2021) established that higher inoculum concentrations of Φ6 suspended in saline solution versus LC medium had varying protective effects. These studies emphasize the role of inoculum suspension matrices in virus inactivation.

An additional limitation of this study is its focus on a 10 s exposure time to reflect typical usage; however, longer exposures which may be necessary for inactivating the highly resistant non-enveloped bacteriophage MS2, were not explored. Moreover, our in vitro suspension experiments may not fully replicate the inactivation dynamics of viruses on hands. Despite these limitations, to our knowledge, this is the first study of the inactivation of enveloped and non-enveloped viruses by foam-based hand sanitizers while considering multiple influencing factors.

Although the CDC recommends applying hand sanitizers for approximately 20 s, a preliminary study (data not shown) indicates that most individuals typically complete this process in about 10 s. While 10 s may be sufficient to inactivate enveloped viruses like SARS-CoV-2, a rubbing time greater than 10 s when using foam-based hand sanitizers will be necessary to achieve a greater log reduction of non-enveloped viruses such as HuNoV. Additionally, while inoculum concentration significantly impacts hand sanitizer efficacy against non-enveloped viruses, the variability among products highlights the need for targeted formulations to ensure effective decontamination across different inoculum levels. Finally, due to the high resistance shown by MS2 to inactivation by the test products, to determine the efficacy of foam hand sanitizers against non-enveloped viruses, MS2 may be the most appropriate and conservative surrogate to utilize in such studies.

## Data Availability

No datasets were generated or analyzed during the current study.
